# The Critical Role of the Branched Chain Amino Acids (BCAAs) Catabolism-Regulating Enzymes, Branched-Chain Aminotransferase (BCAT) and Branched-Chain α-Keto Acid Dehydrogenase (BCKD), in Human Pathophysiology

**DOI:** 10.3390/ijms23074022

**Published:** 2022-04-05

**Authors:** Aikaterini Dimou, Vasilis Tsimihodimos, Eleni Bairaktari

**Affiliations:** 1 Laboratory of Clinical Chemistry, Faculty of Medicine, School of Health Sciences, University of Ioannina, 45110 Ioannina, Greece; kathrine.5@windowslive.com; 2 Department of Internal Medicine, Faculty of Medicine, School of Health Sciences, University of Ioannina, 45110 Ioannina, Greece; tsimiho@gmail.com

**Keywords:** leucine, valine, isoleucine, BCKAs, catabolism, catabolic enzymes, T2DM, heart failure, cancer

## Abstract

Branched chain amino acids (BCAAs), leucine, isoleucine and valine, are essential amino acids widely studied for their crucial role in the regulation of protein synthesis mainly through the activation of the mTOR signaling pathway and their emerging recognition as players in the regulation of various physiological and metabolic processes, such as glucose homeostasis. BCAA supplementation is primarily used as a beneficial nutritional intervention in chronic liver and kidney disease as well as in muscle wasting disorders. However, downregulated/upregulated plasma BCAAs and their defective catabolism in various tissues, mainly due to altered enzymatic activity of the first two enzymes in their catabolic pathway, BCAA aminotransferase (BCAT) and branched-chain α-keto acid dehydrogenase (BCKD), have been investigated in many nutritional and disease states. The current review focused on the underlying mechanisms of altered BCAA catabolism and its contribution to the pathogenesis of a numerous pathological conditions such as diabetes, heart failure and cancer. In addition, we summarize findings that indicate that the recovery of the dysregulated BCAA catabolism may be associated with an improved outcome and the prevention of serious disease complications.

## 1. Introduction

Branched chain amino acids (BCAAs), valine (Val), leucine (Leu) and isoleucine (Ile), are essential amino acids that cannot be synthesized by animals, but only from bacteria, plants and fungi and must be obtained from diet sources. These amino acids have branched hydrophobic side chains and play a primary role in protein structure [1]. All three BCAAs account for approximately 20–25% of most dietary proteins and constitute about 35% of the essential amino acids in mammals [2]. BCAAs and especially leucine are widely known for the anabolic effect on protein metabolism either by promoting muscle protein synthesis or by preventing its breakdown or both, through the activation of the mTOR signaling pathway [3,4]. In addition to the effects on energy metabolism, fatigue and muscle damage during exercise, BCAA supplementation may also have beneficial effects on nutritional status and muscle wasting disorders in many pathological conditions such as liver and kidney diseases and cancer cachexia [5,6,7]. They also act as important nutrient signals and metabolic regulators on glucose homeostasis, neurotransmission, immune response, intestinal development, mitochondrial biogenesis and on milk production from mammary glands [8]. Despite the evidence that BCAA supplementation or intake from a BCAA-rich diet improves metabolic health, several studies highlight their potential role on the pathogenesis and progression of metabolic disorders such as obesity and diabetes or pathological conditions such as heart failure, cancer and neurodegenerative disorders, e.g., Alzheimer disease [9].

## 2. BCAA Catabolism

**Figure 1:**
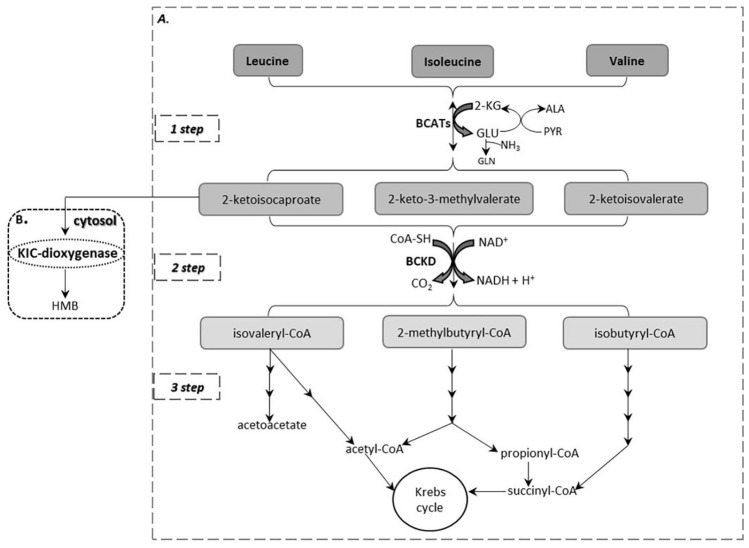
(**A**) BCAA catabolism. (**B**) An alternative pathway of leucine catabolism in the cytosol of the liver.

The first step in BCAA catabolism is a reversible transamination process in which the BCAAs are converted to their branched-chain α-keto acids (BCKAs), 2-ketoisocaproate (KIC), 2-keto-3-methylvalerate (KMV), 2-ketoisovalerate (KIV) from leucine, isoleucine and valine, respectively (Figure 1A). This reaction is catalyzed by branched-chain aminotransferases (BCATs) that exist in two isoforms: the cytosolic (BCATc or BCAT1) that is expressed in the brain and in immune cells, such as activated T lymphocytes and macrophages and the mitochondrial (BCATm or BCAT2) which is found in most tissues, mainly in the skeletal muscle, kidney, pancreas, stomach and colon [10,11,12]. In contrast to most amino acids, the initial site of BCAA catabolism is the skeletal muscle and not the liver due to low hepatic activity of BCAT. The main acceptor of the BCAA amino group, in the process catalyzed by BCAT, is 2-ketoglutarate (2-KG) which yields glutamate (GLU). Then, the amino group can be transferred to pyruvate (PYR) to form alanine (ALA) or another addition of an amino group to glutamate yields glutamine (GLN), as an ammonia detoxification pathway. ALA, GLN and BCKAs are released from muscles into systemic circulation.

**Figure 2:**
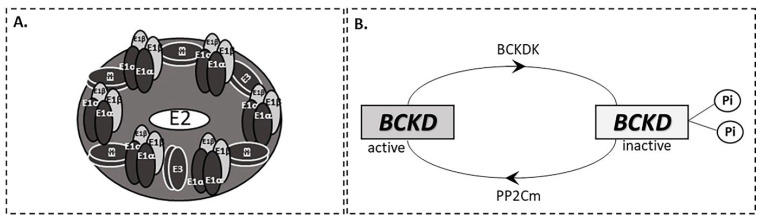
**(A**) BCKD complex. (**B**) Regulation of the BCKD complex.

The second step in BCAA catabolism is the irreversible oxidative decarboxylation of the α-ketoacids to their respective branched-chain acyl-CoA esters, CO_2_ and NADH. By this process, the carbon skeletons of KIC, KMV and KIV are converted to isovaleryl-CoA, 2-methylbutyryl-CoA and isobutyryl-CoA, respectively (Figure 1A). This reaction is catalyzed by the branched-chain α-ketoacid dehydrogenase (BCKD), a multienzyme complex located on the inner mitochondrial membrane which consists of three catalytic components: a thiamine-dependent heterotetrameric (α_2_β_2_) decarboxylase (E1) that catalyzes the oxidative decarboxylation of the BCKAs, a dihydrolipoyltransacylase (E2) to transfer the acyl groups to CoA and a FAD-dependent dihydrolipoyl dehydrogenase (E3) to transfer the released electrons to NAD^+^ [10] (Figure 2A). The activity of the BCKD complex is regulated by the reversible phosphorylation of the E1α subunit; phosphorylation by a specific kinase (BCKD kinase, BCKDK) leads to inactivation while dephosphorylation by a specific phosphatase (protein phosphatase 2Cm, PP2Cm) leads to activation (Figure 2B). This specific kinase, that is the main regulator of this complex activity, is allosterically suppressed by BCKAs with the greatest affinity for KIC [13]. Moreover, efficient inhibition in the BCKD enzyme activity is mediated by increased ratios of NADH/NAD^+^ and of acyl-CoA/CoA-SH as well as ATP [2]. The BCKD enzyme is most active in the liver, intermediately in kidneys and heart and relatively low in muscle, adipose tissue and brain [2]. As a consequence, the BCAA degradation process occurs mainly in muscles and liver compared to other tissues.

The third step in BCAA catabolism leads to ATP production through separate pathways for each amino acid. Catabolism of KIC generates acetyl-CoA and acetoacetate; thus, leucine is considered to be a ketogenic amino acid. KIV is catabolized to succinyl-CoA classifying valine as a glucogenic amino acid. Finally, isoleucine is considered as both glucogenic and ketogenic, because KVM degradation yields succinyl-CoA and acetyl-CoA. An alternative pathway of leucine catabolism has been described in the cytosol of the liver that involves the oxidation of KIC to 3-hydroxy-3-methylbutyrate (HMB) by the enzyme 2-ketoisocaproate dioxygenase (KIC dioxygenase) [14] (Figure 1B). 

## 3. Intracellular Signaling of BCAAs: The Mechanistic Target of Rapamycin (mTOR) Signaling Pathway

BCAAs and especially leucine induce a number of metabolic and signaling functions, particularly via the activation of the “so called” mechanistic Target Of Rapamycin (mTOR) signaling pathway. mTOR is a protein kinase and acts as a central regulator of many fundamental cell processes from protein synthesis to autophagy and glucose homeostasis, while impaired mTOR signaling has been involved in the progression of pathological conditions such as cancer and diabetes as well as in aging [15]. mTOR is a catalytic subunit of two structurally and functionally distinct complexes, mTORC1 and mTORC2 [11]. mTORC1 promotes protein synthesis and regulates autophagy while mTORC2 functions as an effector of the insulin/PI3K signaling pathway [15].

**Figure 3:**
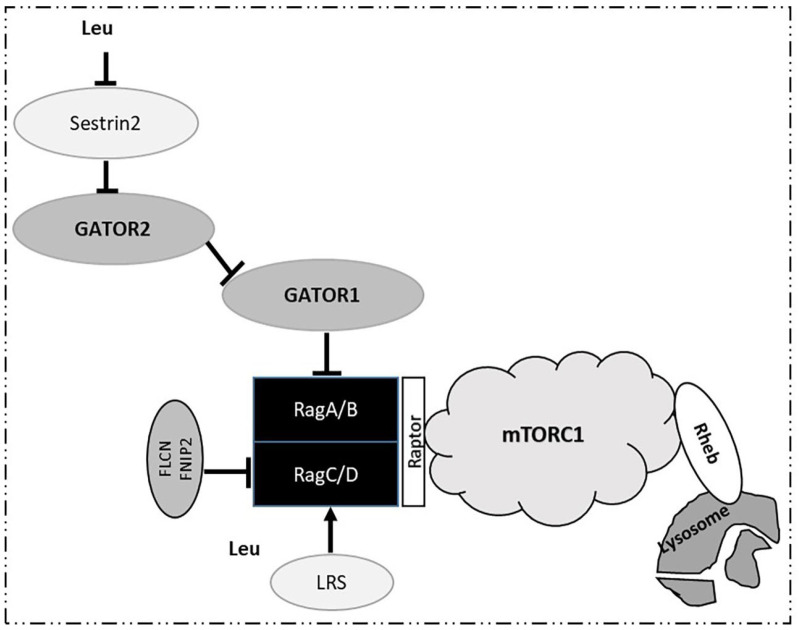
Activation of mTORC1 by leucine.

Increased intracellular levels of amino acids and especially leucine, promote the activation of mTORC1 through the active complex of Rag guanosine triphosphatases (GTPases) consisting of GTP-bound RagA/B and GDP-bound RagC/D [16] (Figure 3). The binding of GTP-RagA/B to Raptor (regulatory associated protein of TOR), promotes the translocation of mTORC1 to the lysosome, where the activated GTP-binding protein Rheb (Ras homolog enriched in brain) stimulates the kinase activity of mTORC1 [17]. The negative regulation of Rag GTPases is mediated by two other complexes, the GATOR1 for Rag A/B and Folliculin-FNIP2 (folliculin interacting protein 2, FNIP2) for Rag C/D. GATOR1 inhibits mTORC1 signaling by acting as a GAP (GTPase-activating protein) on Rag A/B (Figure 3). In addition, GATOR2, another pentameric complex, is a positive regulator of mTORC1 signaling through its interaction with GATOR1 at the lysosomal membrane. Moreover, it is reported that Sestrin2, which is a GATOR2 interacting protein, can inhibit mTORC1 signaling under amino acid deprivation. Across this entire pathway, leucine activates mTORC1 through its direct binding to the leucine sensor, Sestrin2 [18].

Moreover, leucyl-tRNA synthetase (LRS) functions as another leucine sensor for mTORC1 signaling. LRS is translocated to lysosomes where it acts as a GAP promoting the formation of GDP-RagC/D by the GTP hydrolysis of RagC/D, leading to mTORC1 activation (Figure 3). Thus, LRS through the mTORC1 signaling pathway functionally regulates autophagy [19]. Of note, leucine induces a much stronger impact on mTORC1 than the other amino acids. Depletion of leucine alone is as effective as total amino acid starvation in the suppression of mTORC1 signaling, while stimulation with leucine alone is sufficient to promote mTORC1 signal transduction [20].

## 4. BCAAs in Diseases

### 4.1. Inborn Errors of BCAA Metabolism

**Figure 4:**
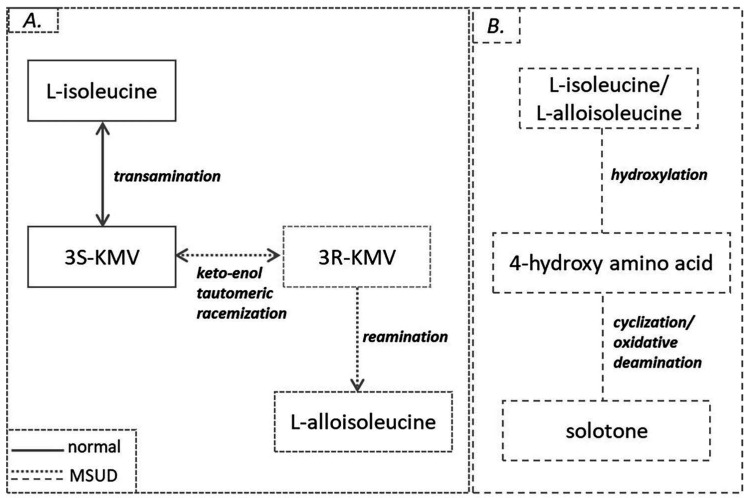
(**A**) L-alloisoleucine formation. (**B**) Solotone formation in MSUD.

Maple syrup urine disease (MSUD) or branched chain α-ketoacid dehydrogenase deficiency, is an autosomal recessive disorder, caused by mutations in the subunits of the BCKD complex (Figure 2A) and occurs in approximately 1:200,000 births [21,22]. Classic MSUD arises from biallelic mutations in the E1α, E1b or E2 subunits. Mutations in the E3 subunit contributes to the activation of pyruvate dehydrogenase and a-ketoglutarate dehydrogenase and leads to a more severe phenotype of MSUD that is characterized by lactic acidosis and progressive neurologic worsening [23]. The biochemical hallmark of the disease is the increased levels of plasma BCAAs and plasma and urine BCKAs. As a result of inactive BCKD, in plasma of MSUD patients racemic KMV (3S, 3R) and alloisoleucine that approaches the levels of isoleucine (Figure 4A) have been detected. It is suggested that a keto-enol tautomeric racemization is the route by which L-isoleucine is slowly converted into L-alloisoleucine in vivo in MSUD patients. Thus, 3S-KMV, produced by the transamination of L-isoleucine and it is unusually accumulated in these patients yields 3R-KMV, which can be further reaminated to alloisoleucine. L-alloisoleucine cannot be transaminated back to 3R-KMV, excreted in the urine or used for protein synthesis [24]. As a consequence, elevated plasma L-alloisoleucine is the pathognomonic disease marker [25]. Accumulation of a rare catabolic product called solotone is responsible for urine’s characteristic odor in MSUD (Figure 4B). Solotone is probably produced by the excess isoleucine or alloisoleucine and is formed through a series of competing reactions with L-isoleucine’s decarboxylation, including hydroxylation, cyclization and oxidative deamination [26].

Current management for MSUD patients includes dietary modifications with limited protein consumption and BCAA-free medical formulas and in some cases liver transplantation to restore BCKD activity and BCAA homeostasis [21]. Disruption of BCAA catabolism may drive neurologic dysfunction in MSUD patients, mainly due to the brain-specific role of these amino acids. One mechanism by which BCAA accumulation contributes to brain toxicity is associated with the biosynthesis of neurotransmitters. BCAAs and especially leucine competes with other large neutral amino acids for transport across the blood–brain barrier [27]. Some of these amino acids such as phenylalanine and tyrosine are important neurotransmitter precursors and as a consequence the competition for transport affects neurotransmitter synthesis. Within the brain, BCAA transamination (Figure 1A) supplies a significant amount of nitrogen for the formation of glutamate, the major excitatory neurotransmitter, and contributes to the maintenance of nitrogen homeostasis in astrocytes and neurons [28]. It is also reported that elevated KIC levels in MSUD may reflect glutamate depletion by increasing the rate of its oxidation [29]. In addition, the accumulation of BCAAs and BCKAs may contribute to neurotoxicity by the stimulation of lipid peroxidation and the induction of oxidative stress [30]. In this condition, carnitine supplementation that is considered as an efficient antioxidant is thought to be beneficial for neurocognitive outcomes [31]. Another mechanism contributing to toxicity is the disruption of brain energy metabolism. The accumulation of leucine and BCKAs inhibits the function of the enzymes pyruvate dehydrogenase and α-ketoglutarate dehydrogenase, as well as the mitochondrial respiration chain [21].

BCAA homeostasis is of major importance for brain function as the accumulation of BCAAs and of toxic BCKAs is associated with severe neurologic diseases. As a consequence, a better understanding of the mechanisms that connect BCAA metabolism with the central nervous system may provide novel therapeutic approaches in MSUD.

### 4.2. Type 1 Diabetes Mellitus (T1DM)

It has been proven repeatedly that in animal models of T1DM [32,33,34] and in patients with T1DM [35,36], the plasma BCAAs are significantly elevated compared to healthy controls. Untreated T1DM is characterized by hyperglycemia, increased food intake, increased fatty acid oxidation as an energy source, muscle wasting and muscle mitochondria dysfunction [37,38]. In this condition, the enhanced muscle proteolysis which greatly exceeds the BCAA oxidation capacity may result in their inadequate transamination and catabolism and subsequently lead to elevated plasma levels of these metabolites [39]. It is suggested that the underlying mechanism involves disruptions in glycolysis and fatty acid oxidation. In T1DM, a decreased rate of glycolysis, reduced activity of citric acid enzymes and increased NADH/NAD^+^ ratio due to enhanced fatty acid oxidation may contribute to the impaired BCAA catabolism as a consequence of the decreased supply of amino group acceptors (2-KG, PYR, oxaloacetate) and the inhibitory effect of NADH and acyl-CoAs on BCKD [32,39].

### 4.3. Obesity, Insulin Resistance (IR) and Type 2 Diabetes Mellitus (T2DM)

Increased plasma BCAAs in obesity and insulin resistance states were reported for the first time in the 1960s [40,41]. These observations have also been confirmed in recent studies, but it is not still clear whether BCAAs are causative factors in the development of insulin resistance (IR), or whether they are biomarkers of impaired insulin sensitivity. Apart from the clear neurotoxic effects of large excesses in BCAAs reported in MSUD patients, the potential pathogenic effects of mild elevation on the levels of these amino acids are slowly being revealed. Multiple mechanisms that contribute to increased BCAAs and subsequently to the pathogenesis of obesity and IR states have been proposed [42,43,44,45].

**Figure 5:**
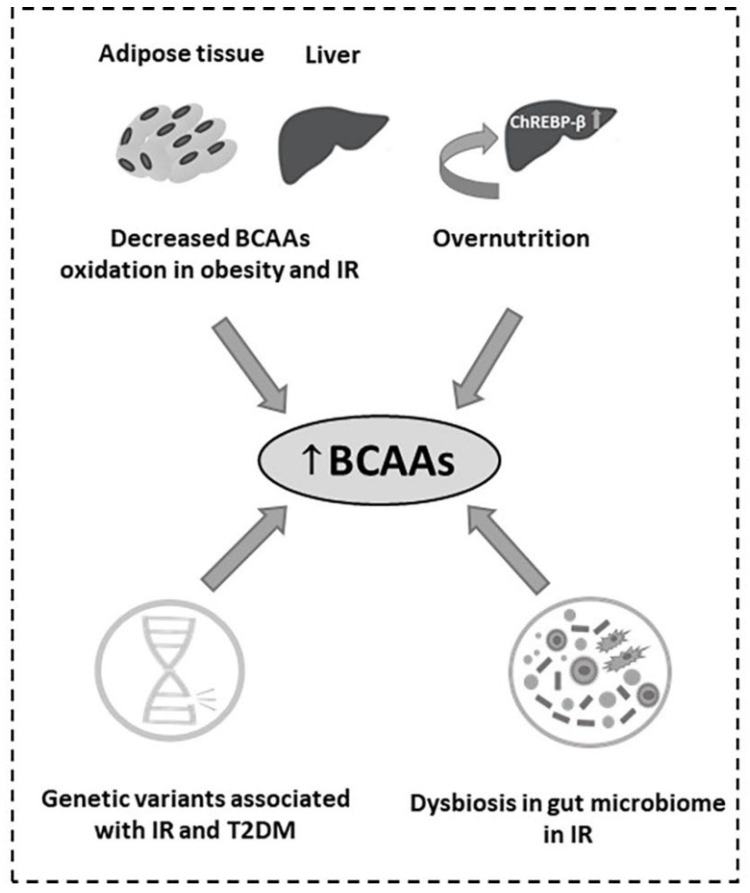
Causes of the (**↑**) increased BCAAs in obesity, IR and T2DM.

In the adipose tissue of obese and insulin resistant humans and animals, the expression of genes encoding the enzymes BCAT2 and BCKD, that catalyze the first two steps of BCAA catabolism as mentioned above, are significantly suppressed [42,46] with hypoxia, inflammation and endoplasmic reticulum stress being the main contributors to this suppression [47,48] (Figure 5). It is reported that in adipose tissue of Zucker-fatty rats and ob/ob mice, the mRNA expression and the enzymatic activity of the above two enzymes was decreased, mainly due to an increased phosphorylation and subsequent suppression of the E1α subunit of BCKD complex compared to the lean controls [49]. In addition, in these obese models, BCKD activity in the liver was diminished whereas in skeletal muscle it was not affected [49,50]. It is proposed that defects in the two key BCAA catabolic enzymes in adipose tissue may play a pivotal role in the higher BCAA levels which accompany obesity, suggesting the adipose tissue as a major contributor to the whole body BCAA catabolism. Moreover, in humans after gastric bypass surgery, plasma BCAAs declined mainly as a result of increased enzymatic activity of BCAT2 and BCKD E1α subunits in both visceral and subcutaneous adipose tissues, indicating a possible role of the adipose depot in restoring the BCAA homeostasis after weight loss [50,51].

In recent years, genome-wide association studies have revealed common genetic variants that correlate with blood amino acid levels. It is reported that mutations in the PPM1K gene in humans and rodents that cause defects in PP2Cm production, the only BCKD phosphatase, result in a defective activation of the BCKD complex [43] (Figure 2). Therefore, BCAA and BCKA levels are similar to those in the mild form of MSUD. Moreover, a common variant rs1440581 near the PPM1K gene was found to be associated with high plasma BCAAs, less weight loss in response to dietary interventions [52], decreased insulin sensitivity and T2DM [53,54] (Figure 5). Furthermore, overnutrition, particularly diets high in fructose, induce the expression of the hepatic transcription factor ChREBP-β by which, levels of the BCKDK are increased and those of PP2Cm decreased, leading to elevated BCKDK:PP2Cm ratio and inhibition of the activity of the BCKD complex, contributing to the obesity-related rise in plasma BCAAs and BCKAs [45] (Figure 5). 

In insulin resistance states, dysbiosis in the human gut microbiome, e.g., gut enriched with the main drivers of the gut bacterial biosynthesis of BCAAs *Prevotella copri* and *Bacteroides vulgatus* and deprivation of genes encoding the transport system for bacterial BCAA uptake, results in increased plasma BCAAs [44] (Figure 5).

The mechanism by which the elevated BCAAs in systemic circulation may cause insulin resistance remains unclear. In fact, it is not well understood whether the increased BCAA levels per se or their disturbed metabolism in various tissues promote insulin resistance.

An elevated hepatic BCKDK:PP2Cm ratio that causes the inactivation of the BCKD complex may also induce the phosphorylation and activation of the ATP citrate lyase (ACL), an important regulator of de novo lipogenesis. ACL promotes increased formation of the lipogenic substrates, acetyl-CoA and malonyl-CoA, which result in the elevated fatty acid synthesis and dyslipidemia that contribute to insulin resistance [45].

**Figure 6:**
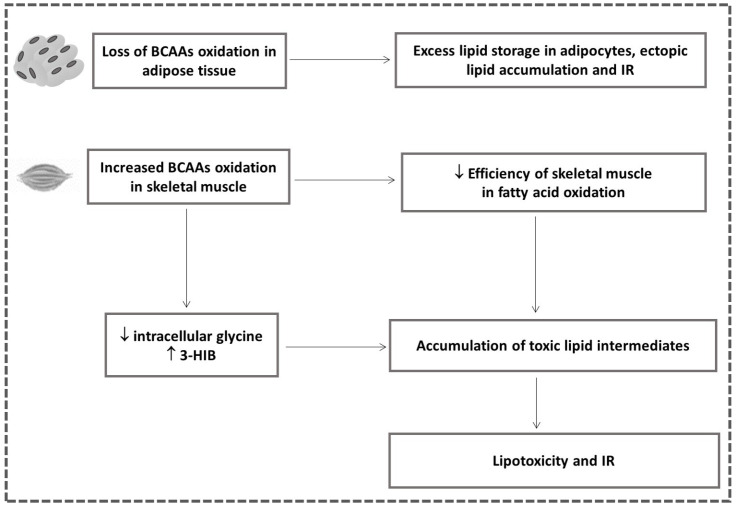
Association between the altered BCAA catabolism in adipose tissue and skeletal muscle with IR (**↓** **downregulated;** **↑ upregulated**).

It has been documented that the activity of BCAAs catabolic enzymes greatly increase over the course of adipocyte differentiation [55,56]. BCAAs contribute significantly to overall lipid synthesis through the generation of the lipogenic substrates, acetyl-CoA and propionyl-CoA. Loss of BCAA oxidation in adipose tissue negatively influences adipogenic differentiation and contributes to excess lipid storage in adipocytes, ectopic lipid accumulation and insulin resistance (Figure 6).

It is proposed that disturbed BCAA catabolism in adipose tissue and liver promote a shift of BCAA flow to skeletal muscle turning this tissue into the main BCAA oxidation site [57]. Several mechanisms have been proposed to connect the increased BCAA oxidation flux in skeletal muscle with the development of insulin resistance. Despite the report that the contribution of BCAAs to total muscle oxidation fuel is much smaller than that of fatty acids [57], one model suggests that an obesity-related increase in BCAA flux in skeletal muscle causes elevated substrate load in mitochondria and decreases the efficiency of skeletal muscle in fatty acid oxidation leading to the accumulation of toxic lipid intermediates [58] (Figure 6).

In addition, an excess of BCAA oxidation in skeletal muscle that depletes intracellular glycine [59] and reduces lipid export of acyl-glycine adducts, promoting the accumulation of the fatty acyl-CoA species suggests a possible link of the high BCAA catabolism in skeletal muscle with obesity-related insulin resistance [58] (Figure 6).

Furthermore, increased BCAA oxidation in skeletal muscle results in the elevated production of 3-Hydroxyisobutyrate (3-HIB), a catabolic intermediate of valine, which acts as a paracrine factor, leads to increased endothelial fatty acid uptake, accumulation of incompletely esterified lipids in skeletal muscle and consequently lipotoxicity and impaired insulin signaling [60] (Figure 6). Moreover, increased plasma 3-HIB has been found in individuals with insulin resistance and it is considered as a marker of future risk of diabetes possessing an important role in the regulation of metabolic flexibility [61]. Excess nutrients and particularly BCAAs may cause insulin resistance due to the activation of the mTORC1, the well-known regulator of skeletal muscle insulin signaling and metabolism [62,63]. BCAA-activated mTORC1 and the consequent activation of serine kinase S6K1 leads to the insulin receptor substrate (IRS)-1 phosphorylation that blocks insulin signaling [62,63]. However, it is not clear if the activation of mTORC1 by BCAAs is necessary and sufficient to induce insulin resistance [62,64].

BCAAs have emerged as predictive markers for future risk of T2DM [65,66] and potential biomarkers of T2DM, as higher plasma BCAAs have been found in animal models and patients with T2DM [67,68,69]. In clinical studies, the elevated levels of BCAAs positively correlate with insulin resistance, HOMA-IR and the levels of HbA1c [70,71]. According to the most prevalent theories, increased BCAAs and especially leucine lead to hyperactivation of mTORC1 and impaired insulin action. As a consequence, the increased demand for insulin along with inflammation and lipotoxicity associated with IR, promote hyperinsulinemia and early exhaustion of beta cells, features characteristic of T2DM [62]. Alternatively, not BCAAs per se, but their impaired catabolism associated with the decreased expression of the genes encoding the catabolic enzymes of BCAAs, results in the accumulation of toxic metabolic intermediates that contribute to the dysfunction of pancreatic beta cell, stress signaling and apoptosis [62]. Moreover, a synergistic action of excess BCAAs and lipids may underlie the transition from obesity to T2DM, as chronic elevations in BCAAs and in circulating fatty acids reinforce the state of chronic hyperinsulinemia and the persistent secretory pressure on the beta cell, contributing to beta cell dysfunction [72]. In T2DM, the elevations in plasma BCAAs are modest when compared to those found in untreated T1DM. Moreover, unlike T1DM, proteolysis does not contribute to the elevated BCAA levels that accompany T2DM [39].

### 4.4. Diabetic Kidney Disease (DKD)

Chronic hyperglycemia leads to long-term complications, such as nephropathy and renal failure that contribute to increased disability and reduced life expectancy in patients with diabetes [73]. It is reported that the lower plasma BCAAs in T2DM patients may indicate a first sign of kidney dysfunction [74]. As mentioned above, the activity of the hepatic BCKD complex is reduced with respect to obesity and/or diabetes and may contribute to increased BCAA levels observed in these conditions. During chronic hyperinsulinemia, which associates with the development of diabetic nephropathy, the BCKD activity is increased, through the insulin-induced dephosphorylation and expression of the BCKD complex [75]. As a result, the lower serum BCAA levels that we found in the patients with diabetic nephropathy compared to T2DM patients without diabetes complications, may indicate enhanced BCAA catabolism (unpublished data). However, more investigation is needed on the underlying mechanisms that contribute to the onset of diabetes complications.

### 4.5. Gestational Diabetes Mellitus (GDM)

Across gestation, the sum of plasma BCAAs decreases while the BCKAs are found either unaltered (2-keto-3-methylvalerate and 2-ketoisovalerate) or decreased (2-ketoisocaproate). In human placental tissue, high activity of BCATs has been detected indicating that BCAAs are transaminated for placental nitrogen demands such as glutamate synthesis [76]. Although increased BCAA levels are strongly associated with insulin resistance and T2DM, studies on plasma of GDM women vs. normal pregnancy have yielded controversial results. In one study, higher serum BCAAs have been observed in high-FPG (fasting plasma glucose) pregnant women compared to low-FPG mothers, suggesting the association of elevated BCAAs with hyperglycemia and insulin resistance [77]. Other studies without significantly elevated BCAA levels in GDM suggest that the elevated ketone bodies in GDM women cause a marked reduction in BCAA catabolism in skeletal muscles; thus, liver becomes the main organ for BCAA oxidation in order to be consumed as substrates for ketogenesis, gluconeogenesis and to a smaller degree for the TCA cycle operation ([78], and unpublished data). The unaltered or even lower levels of BCAAs in women with GDM compared to normal pregnancies may indicate differences in the pathophysiological mechanism that underlie GDM and T2DM. However, further investigation is needed regarding the role of BCAAs in GDM pathogenesis.

### 4.6. Antidiabetic Drugs

In experimental studies, obese and insulin resistant Zucker rats treated with thiazolidinedione (TZD) drugs showed an improvement in insulin sensitivity and in BCAA metabolic pathways in adipose tissue [79]. More specifically, these drugs, which are peroxisome proliferator-activated receptor-γ (PPAR-γ) ligands, reinforce the BCAA catabolic pathway in adipose tissue by restoring the genes that encode BCAA catabolism enzymes [79]. In cultured myotubes, metformin, a common antidiabetic drug, suppresses BCAA catabolic enzymes, possibly leading to BCAA accumulation and reduced production of gluconeogenic precursors; thus helping to control glucose homeostasis [80]. Empagliflozin, a sodium–glucose cotransporter 2 (SGLT2) inhibitor, in addition to its antidiabetic effect, is associated with reduced cardiovascular mortality in patients with T2DM and cardiovascular disease or heart failure. It has been hypothesized that empagliflozin promotes an efficient use of ketone bodies as energy fuel, which may improve myocardial work function. In heart failure, BCAA catabolism is diminished. Empagliflozin may exhibit some of its beneficial effects by restoring BCAA oxidation and by utilizing these amino acids as alternative substrates for ketogenesis [81]. In a previous study, we showed that Dapagliflozin, another SGLT2 inhibitor, leads to increased urine BCAA levels as a result of their decreased proximal reabsorption [80]. Since the incorporation of amino acids is an essential step for proximal tubular cell hypertrophy (an early manifestation of diabetic nephropathy), it can be assumed that reduced BCAA reabsorption prevents proximal tubular hypertrophy. Moreover, altered levels of six BCAA catabolic intermediates after dapagliflozin administration possibly reflect an improved BCAA catabolism and are in line with those observed after empagliflozin administration [82]. These findings suggest that BCAAs respond to therapeutic interventions and improvements in their metabolism may be beneficial for the prevention of diabetes complications.

### 4.7. Heart Failure

BCAA catabolism has also been investigated in cardiovascular disease and heart failure. Higher plasma BCAAs as well as their catabolic products are associated with increased risk of cardiovascular disease [83,84]. Both BCAAs and BCKAs were found elevated in human and in pressure-overload mouse failing hearts [85]. One proposed mechanism suggests that suppression of BCAA catabolic gene expression, which may be regulated by overexpressed Krüppel-like factor 15 in heart failure, results in the intramyocardial accumulation of BCKAs. BCKAs may promote cardiac dysfunction through the suppression of the respiratory chain and the increased release of ROS [85]. In addition, deletion of PP2Cm induces the downregulation of BCAA catabolism leading to elevated circulating and cardiac BCAA levels followed by worse cardiac response to ischemia-reperfusion injury [85]. Moreover, elevated leucine levels may activate mTOR, causing cardiac insulin resistance and hypertrophy, whereas the inhibition of mTOR results in improved cardiac function in heart failure models [86]. The higher BCAAs inhibit both pyruvate and 2-KG dehydrogenases resulting in reduced mitochondrial ATP production and thus contributing to impaired myocardial contractility [87,88]. Additionally, a diet enriched in BCAAs is reported to worsen heart function after myocardial infraction [86]. Pharmacological accentuation of BCAA catabolism reduces plasma and cardiac BCAAs and preserves cardiac function, highlighting the importance of BCAA catabolic efficiency after heart failure [86].

### 4.8. Alzheimer’s Disease (AD)

AD is a chronic neurodegenerative disease and the predominant cause of dementia. It is characterized by extracellular beta-amyloid (Aβ) deposits and intracellular hyperphosphorylated Tau proteins [89]. Recent studies suggest a potential role of BCAAs in pathogenesis of AD, but current evidence does not provide a clear conclusion [90,91]. In experimental animal studies, it is reported that the reduced BCAT1 expression in brain tissues of transgenic AD mice models at certain ages may result in the accumulation of plasma BCAAs [90]. In contrast, in a prospective study including hundreds of participants, lower serum valine levels were associated with an increased risk for AD, impaired cognitive function and faster cognitive decline [92]. According to another study on AD patients, the high serum BCAA, glutamate and BCAT levels were positively correlated with AD severity. These results support the “glutamate excitotoxicity” hypothesis in AD pathogenesis, which is enhanced glutamate production by elevated BCAT activity that may worsen brain functions [93]. Moreover, it is suggested that individuals genetically predisposed to elevated BCAA levels may have an increased susceptibility to AD [94]. This predisposition comes from the identification of several nucleotide polymorphisms in the isoleucine degradation pathway in AD patients, including the gene expression of the specific phosphatase of the BCKD complex. In addition, the excess of BCAAs is associated with the imbalance of key neurotransmitters, with neural oxidative stress and apoptosis and with the mTOR hyperactivation that leads to brain insulin resistance [9]. BCAA-enriched diets may contribute to the development of AD, by the stimulation of the Tau phosphorylation through an mTOR-dependent manner which worsens cognitive performance in AD animal models [90]. In contrast, BCAA restriction improved memory functions in the same animals [95,96]. These findings suggest that overload of BCAAs or their defective catabolism may have a causal role in AD pathogenesis and progression. Further investigation is needed for treatment strategies targeting not only Aβ or Tau proteins but also brain and systemic metabolic abnormalities.

### 4.9. Cancer

Abnormal BCAA levels have also been reported in patients diagnosed with cancer. Increased plasma BCAAs were found in patients 2–5 years before the diagnosis of pancreatic ductal adenocarcinoma (PDAC) and these elevations were associated with an up to two-fold increase in the risk for the development of pancreatic cancer [97]. Subclinical protein breakdown in early tumorigenesis, in order to meet the BCAA demands of the growing tumor, may result in their higher plasma levels [97]. These observations were also confirmed in mice with mutant-KRAS-driven pancreatic tumors, as elevated levels of BCAAs were found before the manifestation of subclinical cancer [98]. However, no alterations were observed in plasma glucose levels, suggesting that the increased BCAAs are not associated with impaired glycemic control or insulin resistance but may indicate an early sign before the manifestation of PDAC. Moreover, it is reported that the metabolic fate of BCAAs is not the same for all tumors, even for those caused by the same mutations [98]. PDAC tumors exhibit reduced BCAAs uptake compared to non-small cell lung carcinoma, which display enhanced BCAAs uptake for protein synthesis [98].

Several studies focus on the activity of BCAT1 in cancer, as alteration in the expression of this enzyme is differentiated among the cancer types and is associated with tumor aggressiveness [99,100]. Transcriptional studies revealed that in glioblastoma tumors, classified according to the isocitrate dehydrogenase (IDH) mutation status, BCAT1 expression was significantly higher in IDH_wt_ gliomas while in IDH_mut_ gliomas it was suppressed [99]. This suppression is mediated by 2-hydroxyglutarate (2-HG) produced from 2-KG by IDH_mut_ enzymes. The oncometabolite 2-HG reduces the activity of 2-KG-dependent enzymes such as histone demethylases, leading to widespread hypermethylation of the BCAT1 promoter region and thus to the BCAT1 suppression [99,100]. Breakdown of BCAT1 limits the supply of glutamate and thereby increases the dependence on glutaminase that is the alternative route for the biosynthesis of glutamate and the antioxidant glutathione. It is reported that inhibition of glutaminase sensitizes the IDH_mut_ glioma cells to oxidative stress in vitro and to radiation in vitro and in vivo, suggesting an effective role of this inhibition against IDH_mut_ gliomas [101].

In leukemia cells, overexpression of BCAT1 decreases the intracellular 2-KG and causes DNA hypermethylation through inactivation of 2-KG-dependent enzymes and especially TET (ten-eleven translocation family of DNA demethylases) [102]. In IDH_wt_ acute myeloid leukemia, higher BCAT1 levels are associated with shorter survival and display a DNA hypermethylation phenotype similar to previously described IDH mutations. These findings suggest that BCAT1 stabilizes HIF1α protein, which is required for the maintenance of leukemia stem cells, by limiting intracellular 2-KG and regulates the epigenomic landscape [102]. In chronic myeloid leukemia (CML), BCAT1 promotes the clonogenic growth by the reamination of BCKAs to form BCAAs and, thus, activating the mTOR pathway. However, knockdown of BCAT1 promotes cellular differentiation and prevents the propagation of blast crisis CML in vitro and in vivo [103].

Tumor tissues of patients with hepatocellular carcinoma (HCC), compared to adjacent non-tumor tissues, displayed a significant increase in BCAAs but a reduction in their oxidized metabolic intermediates, indicating impaired liver BCAA catabolism [104]. Moreover, tumors from HCC patients showed abnormally high gene expression of BCATs but low expression of BCKD and the downstream catabolic enzymes [104]. In addition, it is suggested that in HCC tumors, the elevated BCAA levels promote hyperactivation of mTOR signaling for tumor growth rather than being oxidized for energy demands. Interventions in the media of cultured HCC cells such as decreased content in BCAAs or enrichment with the mTOR inhibitor rapamycin or inhibition of BCKDK result in lower tumor cell proliferation rates [104].

In contrast, breast cancer displays enhanced BCAA catabolism with higher gene expression of BCAT1, BCKD and other downstream enzymes [105]. Thus, in these tumors, BCAT1 knockdown may contribute to reduced mTOR signaling and, as a consequence, a decreased growth rate of breast cancer cell lines [105].

These findings indicate the major role of BCAT1 in tumor proliferation and that its expression in different cancer types may possibly serve as a therapeutic target. In addition, in the presence of cachectic disorder in cancer patients, supplementation of BCAAs improves the quality of life, due to their anabolic effect, and subsequently the effectiveness of the chemotherapeutic interventions [106].

### 4.10. Liver and Kidney Disease

Decreased BCAAs and elevated aromatic amino acids represent characteristic alterations in blood of cirrhotic patients along with an increased risk of hepatic encephalopathy and muscle wasting disorders [107]. The lead causes are: (1) the increased catabolic rate of BCAAs to promote the formation of glutamate as in hyperammonemia states [108], (2) the heightened uptake of BCAAs by muscles in chronic malnutrition and (3) to a smaller degree their increased oxidation to be used as gluconeogenic precursors [109].

In hepatitis B, disturbed amino acid metabolism, expressed mainly as increased tyrosine and decreased BCAA levels, results in a significantly lower ratio of BCAAs to tyrosine (BTR) that is correlated with the type and stage of HBV infection [110]. BTR can also be used to determine the degree of liver fibrosis in patients with hepatitis C virus (HCV) infection and is improved along with liver fibrosis after interferon-alpha treatment [111].

Elevated plasma BCAA levels have been recorded in patients with nonalcoholic fatty liver disease (NAFLD); however, it remains unknown if this is due to increased muscular proteolysis, obesity, and/or increased insulin resistance or impaired liver and adipose tissue catabolism of these amino acids [112]. Furthermore, it has been proposed that NAFLD and disturbed BCAAs catabolism in this condition could have a synergistic effect on the development of T2DM [113].

BCAA supplementation may have beneficial effects in patients with chronic liver diseases. In cirrhotic patients, it improves the nutritional status, weakness and fatigue and reduces the incidence of hepatic encephalopathy and HCC [114]. Moreover, supplementation with BCAA formulas contributes to glucose homeostasis, to a better immune system function and in the rise in serum albumin levels [114]. In chronic hepatitis C, BCAA supplementation may upregulate interferon signaling (a potent antiviral agent for chronic HCV infection) through mTOR activation [115]. In addition, BCAAs stimulate the intestinal immunoglobulin A secretion, which improves the mucosal surface defense affected by HCV [116]. Moreover, valine administration is associated with the reduction in HCV load, by improving the function of monocyte-derived dendritic cells and by the activation of interferon signaling [117].

Finally, in chronic renal failure, plasma BCAAs and BCKAs as well as valine levels in muscles are found significantly decreased [118,119]. In a cohort study with stages I and II chronic kidney disease (CKD) patients, plasma valine and leucine but not isoleucine were significantly lower compared to healthy controls, suggesting the potential role of BCAAs as biomarkers of the early stage of the disease [120]. Malnutrition, hemodialysis, metabolic acidosis and systemic inflammation response in CKD patients may contribute to the decreased BCAA levels [121]. More specifically, metabolic acidosis and systemic inflammation contribute to accelerated proteolysis and enhanced BCKD activity resulting in the increased use of BCAAs as substrates for the synthesis of acute phase proteins and proteins of the immune system [122,123]. BCAA formulas together with other essential amino acids are provided to patients with chronic renal failure in order to maintain protein balance and to minimize uremic toxicity [5].

## 5. Conclusions

Recent approaches have emphasized the role of BCAAs not only as important constituents for protein synthesis but also as important regulators of whole-body metabolism. BCAA-rich diets may have a beneficial role in metabolic health, mainly due to their anabolic effect, but further investigation is needed regarding the relationship of elevated BCAAs with a wide range of pathological conditions. Apart from the well-characterized Mendelian disorders, defective BCAA catabolism is linked to the pathogenesis and progression of many prevalent diseases. A better understanding of the mechanisms underlying the role of BCAAs in these pathological conditions may be helpful and may lead to better outcomes possibly by “restoring” BCAA metabolism.
